# Effects of Temperature, Salinity and Fish in Structuring the Macroinvertebrate Community in Shallow Lakes: Implications for Effects of Climate Change

**DOI:** 10.1371/journal.pone.0030877

**Published:** 2012-02-29

**Authors:** Sandra Brucet, Dani Boix, Louise W. Nathansen, Xavier D. Quintana, Elisabeth Jensen, David Balayla, Mariana Meerhoff, Erik Jeppesen

**Affiliations:** 1 Department of Bioscience, Aarhus University, Silkeborg, Denmark; 2 European Commission, Joint Research Centre, Institute for Environment and Sustainability, Ispra, Italy; 3 Institute of Aquatic Ecology and Department of Environmental Sciences, Facultat de Ciències, University of Girona, Campus de Montilivi, Girona, Spain; 4 Departamento de Ecología y Evolución, Centro Universitario Regional Este (CURE)-Facultad de Ciencias, Universidad de la República, Maldonado, Uruguay; 5 Greenland Climate Research Centre (GCRC), Greenland Institute of Natural Resources, Nuuk, Greenland; 6 Sino-Danish Educational and Research Centre, Beijing, China; Institute of Marine Research, Norway

## Abstract

Climate warming may lead to changes in the trophic structure and diversity of shallow lakes as a combined effect of increased temperature and salinity and likely increased strength of trophic interactions. We investigated the potential effects of temperature, salinity and fish on the plant-associated macroinvertebrate community by introducing artificial plants in eight comparable shallow brackish lakes located in two climatic regions of contrasting temperature: cold-temperate and Mediterranean. In both regions, lakes covered a salinity gradient from freshwater to oligohaline waters. We undertook day and night-time sampling of macroinvertebrates associated with the artificial plants and fish and free-swimming macroinvertebrate predators within artificial plants and in pelagic areas. Our results showed marked differences in the trophic structure between cold and warm shallow lakes. Plant-associated macroinvertebrates and free-swimming macroinvertebrate predators were more abundant and the communities richer in species in the cold compared to the warm climate, most probably as a result of differences in fish predation pressure. Submerged plants in warm brackish lakes did not seem to counteract the effect of fish predation on macroinvertebrates to the same extent as in temperate freshwater lakes, since small fish were abundant and tended to aggregate within the macrophytes. The richness and abundance of most plant-associated macroinvertebrate taxa decreased with salinity. Despite the lower densities of plant-associated macroinvertebrates in the Mediterranean lakes, periphyton biomass was lower than in cold temperate systems, a fact that was mainly attributed to grazing and disturbance by fish. Our results suggest that, if the current process of warming entails higher chances of shallow lakes becoming warmer and more saline, climatic change may result in a decrease in macroinvertebrate species richness and abundance in shallow lakes.

## Introduction

Recent studies have shown cold and warm shallow freshwater and brackish lakes have substantially different trophic structure and dynamics [1]–[3]. The higher temperature at lower latitudes is associated with a shift to a fish community dominated by omnivorous fish, with higher specific metabolic and excretion rates, more frequent and earlier reproduction and smaller sizes than at higher latitudes [4]–[6]. The higher densities of small fish, which tend to aggregate among the macrophytes [5], often exert a high predation pressure on the zooplankton and thus reduce grazing on phytoplankton, with implications for the clear water state of warm shallow lakes [7]–[9]. Fish may also consume plant-associated macroinvertebrate grazers and indirectly enhance periphyton growth with cascading effects on plant growth and thus on water clarity, at least in temperate lakes [10]. However, there is little information on how the macroinvertebrate community responds to the potentially increased fish predation in warmer climates. Studies in subtropical shallow lakes [1] have shown lower taxon richness and significantly lower densities of plant-associated macroinvertebrates compared to similar temperate lakes, presumably as a result of the high densities of fish occurring within the macrophytes. In contrast, experimental studies along a latitudinal gradient in Europe [11] showed that fish affected the composition of the plant-associated macroinvertebrate community rather than their abundance, and that the outcome of the interaction depended greatly on the climatic conditions determining the life history traits of the macroinvertebrates [11], [12]. Furthermore, Miracle et al. [11] found the fish effect to depend strongly on the diet of the species involved. As for zooplankton, the decrease in the abundance of plant-associated macroinvertebrate grazers at higher temperatures may have implications for the ecological status of lakes since it may enhance periphyton growth and indirectly promote the turbid water state by outshading macrophytes [13]. In addition, potentially higher predation by small fish within the plants beds may influence the spatial distribution and diel movements of macroinvertebrates. Thus, zooplankton have been shown to migrate to deeper layers or into submerged plants during the day as a result of the tradeoffs between predation risk, food availability and oxygen concentration (reviewed by [14]). However, available information about diel movement of macroinvertebrates is scarce.

There is also emerging evidence that trophic structure changes along a salinity gradient [3], [15]–[17]. Presumably, eutrophic brackish lakes resemble warm freshwater lakes in that they are often turbid and the biomass of zooplankton is lower than in cold freshwater lakes [15], [18]. This has been attributed to a salinity-induced increase in the predation on large-bodied zooplankton and the loss of keystone species such as *Daphnia*, leading to dominance of smaller and less efficient grazer species [3], [16], [19]. The effects of salinity on the community structure of macroinvertebrates are, however, less clear. At salinity ranges from 1 to 3, macroinvertebrate assemblages exhibit reduced species richness and abundance and a change in species composition [20], [21]. Some studies have shown that Crustacea are the most salinity tolerant of the major invertebrate taxon groups [22], [23], whereas Ephemeroptera are among the least tolerant invertebrates [24], [25].

With climate warming we can expect changes in the trophic structure and biodiversity of shallow lakes as a combined effect of increased temperature and salinity, the latter particularly in arid, semiarid and coastal areas [3], [17]. An increased strength of trophic interactions, not least predation, with warming has also been suggested [3], [26]. Cross-comparison studies of shallow lakes in regions differing in temperature may help identify the impacts of these changes, while the comparison of lakes along a salinity gradient may help elucidate the effects of salinity changes. In the present study, we investigated the potential effects of contrasting temperature, salinity (along a short salinity gradient) and fish on the plant-associated macroinvertebrate community structure by comparing similar shallow lakes located in two regions of contrasting temperature regime with salinities corresponding to ca. freshwater (0.3 salinity) to oligohaline waters (3.8 salinity). For this purpose, we performed a field experiment with artificial plants (to control for habitat complexity) in eight shallow lakes in cold temperate (Denmark) and Mediterranean (Spain) regions. We undertook day and night-time sampling of macroinvertebrates associated with the artificial plants. Additionally, we sampled fish and large macroinvertebrate predators in littoral and pelagic areas to investigate their influence on the plant-associated macroinvertebrate community. In the same experiment, we found zooplankton size structure and composition to be highly affected by fish predation [3]. In the present study, we hypothesized that, due to higher fish densities in the warm climate, macroinvertebrate densities would be lower than in the cold climate and that macroinvertebrate richness would decrease with increasing salinity. We also hypothesized that the density and distribution of fish would shape the diel distribution of plant-associated macroinvertebrates, which would probably show a diel distribution reverse to that of their predators.

## Materials and Methods

### Ethics statement

All necessary permits were obtained for the described field studies through the authority responsible at each location (the Natural Reserve Area of Vejlerne in Denmark and the Natural Park of Aiguamolls de l'Empordà in Spain).

### Experimental design

The experiment was carried out in 4 cold temperate shallow coastal lakes located in the north of Denmark and in 4 Mediterranean shallow coastal lakes located in north-east Spain. Both Spain and Denmark belong to the temperate mesothermal climate region, but they have different climates according to the Köppen Climate Classification System. In Spain, the lakes were located in Catalonia, which has a semi-arid climate characterised by hot and dry summers and cool and wet winters (average air temperature 15–16°C). Denmark has a moist continental climate with milder summers and colder and wetter winters (average air temperature in the region where the lakes were located is 7.5–8.1°C). In both regions, we selected permanent shallow lakes with similar total nutrient concentrations and salinities, ranging from 0.3 to 3.8 during the study period (Table 1). For more details on the limnological characteristics of the lakes see [3]. The dominant submerged macrophyte species were *Potamogeton pectinatus* in the Spanish lakes and *Chara aspera* and *Myriophyllum spicatum* in the Danish lakes.

**Table 1:** Main limnological characteristics of the eight study lakes at the time of the experiment. TP, total phosphorus; TN, total nitrogen.

	Cold temperate				Mediterranean			
	0.3	0.5	1.2	3.8	0.4	0.8	1.6	2.2
	(Lund Fjord)	(Selbjerg)	(Glombak)	(Østerild)	(Salins)	(Sirvent)	(Bassa Coll)	(Ter Vell)
Temperature (°C)	17.3	16.7	15.8	17.1	23.1	20.2	20.6	21.7
Secchi depth (m)	0.3	0.2	0.5	0.4	0.6	0.8	0.5	0.4
TP (mg L^−1^)	0.10	0.16	0.09	0.08	0.13	0.03	0.17	0.32
TN (mg L^−1^)	1.81	3.64	2.21	2.20	0.44	7.20	1.53	0.42

The experiment was conducted in late May and early June in Spain and in July in Denmark. To obtain a similar habitat structure and complexity in both regions and in all lakes we used artificial plants mimicking submerged plants. The plant beds were introduced in the littoral zone of the lakes following the methodology described by Brucet et al. [3]. The plant beds consisted of 1 m diameter plastic rings with an attached net from which the artificial plants hung (hereafter modules). Plants were made of green plastic Christmas tree garlands, which have an architecture resembling that of *Ceratophyllum* or *Myriophyllum* spp., and with a local percent volume inhabited by plants of 49% (PVI, [27]; see picture in [9]). Each module consisted of 100 artificial plants (length 0.75 m) which were held at the surface by two strings attached to two poles. Modules were placed at 0.8 m depth in the littoral zone of the lakes. Before the introduction of the artificial plant beds, natural plants were removed at ca. 3 m distance around the modules.

We introduced 16 modules per lake: 8 modules containing submerged plants (4 for daytime samples and 4 for night-time samples), hereafter termed ‘submerged plants’ or ‘S’, and 8 modules with only poles and no plants (for the sampling of fish and large macroinvertebrate predators in the pelagic), hereafter termed ‘open’ sites or ‘O’. The modules were placed in a randomised design to avoid any bias. Modules were introduced one month before the sampling to allow colonisation of the plants by periphyton and invertebrates.

### Sampling and processing

We collected water samples for the analysis of total phosphorus (TP) and total nitrogen (TN) [28], [29] and measured the Secchi disk depth in open water near the experimental set-up. We sampled all plant-associated macroinvertebrates (herbivores, omnivores and predators) larger than 500 µm day and night (four replicates each) by carefully removing three artificial plants from each plant bed. Samples were taken using a small boat to minimise disturbance. We cut a 10-cm-long piece of each plant between 10 and 20 cm depth to determine the biomass of associated periphyton.

We placed a cylindrical net (1.1 m in diameter and 1 mm mesh size) on the sediment beneath each plant bed and at each open site. The nets were attached with strings to two poles. After approximately 12 hours, we sampled the fish from each submerged plant module and each open site module by quickly pulling the strings and lifting the net up above the water surface. The sampling was repeated at night-time in the other half of the modules. Using the same nets, we also sampled large (>1 mm) macroinvertebrates potentially predators from each submerged plant module and each open site module, hereafter called “free-swimming macroinvertebrate predators”. For fish and free-swimming macroinvertebrate predators we had 4 replicates for day and 4 for night-time at each submerged plant bed and open site. Fish and macroinvertebrates were preserved in 70% ethanol. The discrimination between plant-associated and free-swimming macroinvertebrate predators was based on the sampling method, and not on the species traits. Thus, a species of macroinvertebrate potentially predator may appear in both categories (see also Table 2 for details).

**Table 2:** Plant-associated and free-swimming macroinvertebrate species present in cold temperate and Mediterranean shallow lakes with indication of potentially predatory species (*).

	Cold temperate (40)	Mediterranean (20)	*(continues)*	Cold temperate	Mediterranean
Polychaeta	(0)	(1)	Corixidae undet. (P+F)*??	+	
*Nereis diversicolor* (P)*		+	*Microvelia pygmaea* (F)*		+
Hirudinea	(6)	(0)	*Microvelia reticulata* (P+F)*	+	
*Erpobdella octoculata* (P)*	+		*Sigara dorsalis* (F)*		+
*Glossiphonia heteroclita* (P+F)*	+		*Sigara laterales* (F)*		+
*Glossiphonia concolor* (P+F)*	+		Ephemeroptera	(4)	(1)
*Helobdella stagnalis* (P+F)*	+		* Baetis* sp. (P)	+	
*Piscicola geometra* (P)	+		* Caenis luctuosa* (P)	+	
*Theromyzon tessulatum* (P)	+		* Cloeon dipterum* (P)	+	
Oligochaeta	(1)	(1)	* Cloeon inscriptum* (P)	+	+
Tubificidae undet (P)	+	+	Coleoptera	(3)	(3)
Gastropoda	(6)	(2)	* Enochrus* sp. (F)*		+
*Anisus spirorbis* (P)		+	* Gyrinus* sp. (P+F)*	+	
*Bithynia tentaculata* (P)	+		* Halipus* sp. (P)	+	
*Bythina* sp. (P)	+		* Helochares* sp. (F)*		+
*Gyraulus laevis* (P)	+		* Hydroglyphus* sp. (F)*		+
*Potamopyrgus jenkinsi* (P)	+		Hydrophilidae undet. (P)*	+	
*Physella acuta* (P)		+	Trichoptera	(9)	(0)
*Radix balthica* (P)	+		* Athripsodes* sp. (P)	+	
*Valvata piscinalis* (P)	+		* Cyrnus* sp. (P+F)*	+	
Malacostraca	(3)	(4)	* Mystacides longicornis* (P)	+	
*Asellus aquaticus* (P)	+		* Mystacides* sp. (P)	+	
*Atyaephyra desmaresti* (F)*		+	* Oecetis* sp. (P+F)*	+	
*Crangon crangon* (F)*		+	* Oxyethira* sp. (P)	+	
*Gammarus lacustres* (P+F)*	+		* Setodes* sp. (P)	+	
*Leptocheirus pilosus* (P)*		+	* Trichostegia minor* (P+F)*	+	
*Neomysis integer* (P+F)*	+		* Ylodes* sp. (P)	+	
*Procambarus clarkii* (F)*		+	Diptera	(4)	(3)
Odonata	(1)	(2)	Chironomini (P)	+	+
*Aeshna* sp. (F)*		+	Orthocladiinae (P)	+	+
*Coenagrion* sp. (P+F)*	+		Tanypodinae (P+F)*	+	+
*Ischnura elegans* (P)*		+	Tanytarsini (P)	+	
Heteroptera	(3)	(3)			
*Corixa* sp. (P+F)*	+				

(P) macroinvertebrates sampled by removing the artificial plants and therefore considered plant-associated macroinvertebrates; (F) macroinvertebrates sampled by nets and therefore considered free-swimming macroinvertebrates; (P+F) macroinvertebrates sampled both by nets and removing the artificial plants and therefore considered both plant-associated and free-swimming macroinvertebrates. Note: only potentially predators were considered in the free-swimming macroinvertebrate community (see also methods for details). In brackets, number of species for each taxonomic group in each region.

In the laboratory, macroinvertebrates were counted and identified to at least family level and classified as potentially predatory or not predatory according to [30]–[32]. We counted, measured and identified fish to species level and we indicated if they typically consume macroinvertebrates and periphyton according to the literature [33]–[40]. We assessed the data on plant-associated macroinvertebrates and periphyton per unit of plant-covered area. We estimated the density of fish and free-swimming macroinvertebrate predators per unit of area covered by the cylindrical net. We estimated periphyton biomass as chlorophyll-a concentration according to Jespersen and Christoffersen [41]. Macroinvertebrate richness was calculated as the sum of taxa in each treatment.

### Statistical analysis

We used a two-way nested ANOVA to test differences in plant-associated macroinvertebrates between regions and lakes. The factors were: ‘region’ (two levels, cold temperate and Mediterranean) and ‘lake’ (four levels), nested within region. To assess differences in fish and free-swimming macroinvertebrate predators we used a three-way nested ANOVA, with ‘habitat’ (two levels, ‘S’ and ‘O’) as an additional factor.

To identify relationships between plant-associated macroinvertebrate community structure and salinity and the density of potential predators (fish) we used multiple regression. As independent variables we used salinity and mean density of fish per habitat. Response variables in each multiple regression were the density and the richness of plant-associated macroinvertebrates. The condition index was never higher than 10 and tolerance never higher than 0.2, suggesting that multicollinearity between predictory variables was low.

Diel distribution patterns were analysed using a one-way ANOVA [factor time, two levels (‘D’ and ‘N’)] in each lake for plant-associated macroinvertebrates and a two-way ANOVA [factor time, two levels (‘D’ and ‘N’) and factor habitat, two levels (‘S’ and ‘O’)] in each lake for fish and free-swimming macroinvertebrate predators. We assessed whether the night-time densities of organisms differed compared to daytime (i.e. a significant effect of ‘time’ in the ANOVA, suggesting diel movement). For fish and free-swimming macroinvertebrate predators, we also assessed whether the density amongst the submerged plants changed between day and night (significant ‘habitat’×‘time’ interaction in the ANOVA). We log_10_ (x+1) transformed data to fulfil requirements of homoscedasticity and normal distribution of residuals.

## Results

### Community structure at contrasting climates

**Figure 1:**
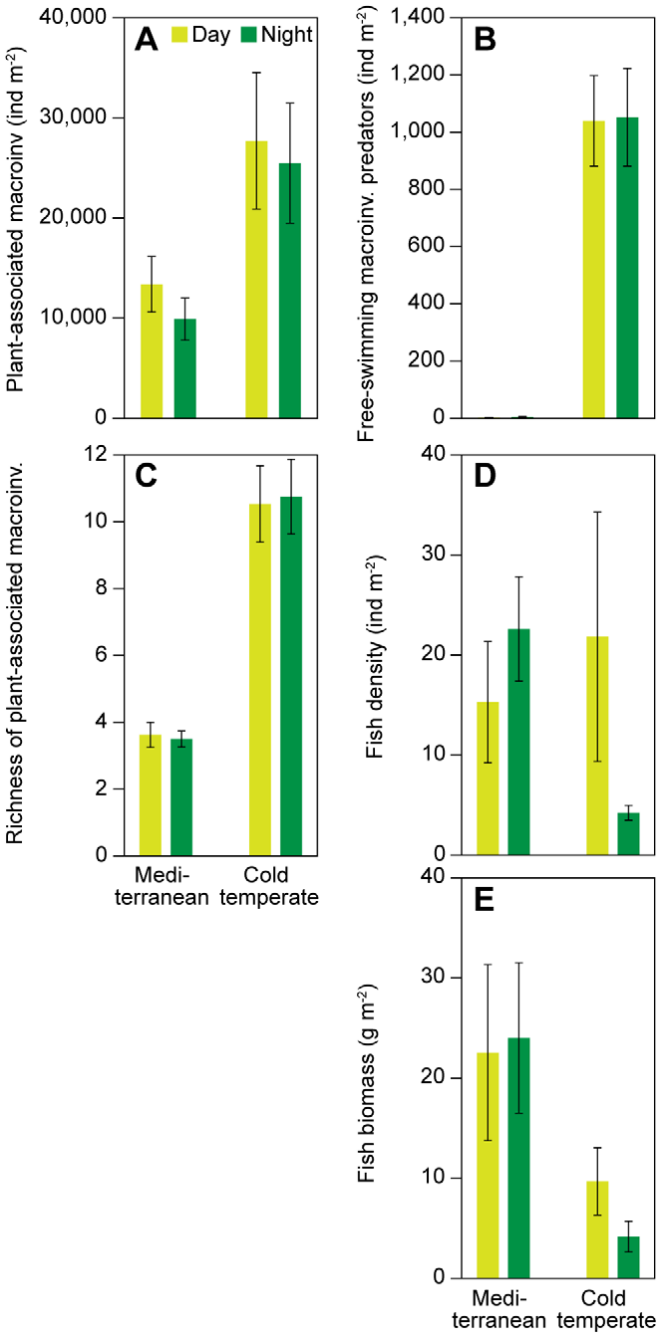
Densities of plant-associated macroinvertebrates, free-swimming potentially predatory macroinvertebrates and fish, and taxon richness of plant-associated macroinvertebrates. The data are means (±1SE) of data collected at day and night-time in four lakes in the cold temperate region and four lakes in the Mediterranean region. Fish biomass in fresh weight.

The density of plant-associated macroinvertebrates was significantly higher in the cold temperate region than in the Mediterranean region (Table 3, Fig. 1). However, apart from region related differences, there were some significant differences among lakes within a region (significant effect of the factor lake in the nested ANOVA, Table 3). When assessing each plant-associated macroinvertebrate taxon group separately, most taxa (7 out of 11) also showed higher abundances in the cold temperate region, except for Malacostraca and Polychaeta, which were more abundant in the Mediterranean region (Table 4), and Gastropoda and Odonata, which had similar densities in both regions. However, in all cases where nested ANOVA was performed, there were also differences among lakes. Diptera was the most abundant plant-associated macroinvertebrate taxon in both regions, closely followed by Oligochaeta in the cold temperate lakes (Table 4). Other abundant taxa were Trichoptera and Malacostraca in the cold temperate region and Malacostraca and Oligochaeta in the Mediterranean region. Taxon richness of plant-associated macroinvertebrates was also higher in the cold temperate region, average richness being 2-fold higher than in the Mediterranean region (Table 3, Fig. 1). A total of 40 macroinvertebrate taxa occurred in the cold temperate lakes, whereas only 20 taxa were found during sampling in the Mediterranean lakes (Table 2). Region related differences were especially remarkable for the densities of free-swimming macroinvertebrates, being scarce in most Mediterranean lakes (Table 3, Fig. 1). Also average periphyton biomass was four times higher in the cold temperate lakes compared to the Mediterranean lakes despite the higher densities of macroinvertebrates and the lower temperature in the former (Table 3).

**Table 3:** Density and richness of plant-associated macroinvertebrates, fish and large free-swimming potentially predatory macroinvertebrates and periphyton biomass in the Mediterranean and cold temperate region.

	Region			Habitat		
	T	M	*p*	O	S	*p*
Density of plant-associated macroinv.	26584 (4474)	10506 (1572)	<0.01	–	–	–
Richness of plant-associated macroinv.	10.9 (0.8)	3.6 (0.2)	<0.01	–	–	–
Free-swim. macroinv. predators	1046 (116)	3.4 (0.9)	<0.01	213 (4.3)	1185 (146)	<0.01
Fish abundance	13.0 (6.3)	19.0 (3.4)	<0.05	2.7 (0.5)	28.0 (7.1)	<0.01
Fish biomass	6.9 (5.7)	23.3 (5.7)	<0.01	1.9 (0.5)	28.2 (5.7)	<0.01
Periphyton	1569 (360)	330 (72)	<0.01	–	–	–

Average (±SE) of density (ind. m^−2^) and richness of plant-associated macroinvertebrates (macroinv.), density of fish and large free-swimming potentially predatory macroinvertebrates (ind. m^−2^) and periphyton biomass (mg m^−2^) and *p*-values for the nested ANOVAs. Two-way nested ANOVA for density and richness of plant-associated macroinvertebrates and periphyton with factors ‘region’ (2 levels, cold temperate ‘T’ and Mediterranean ‘M’) and ‘lake’ (4 levels) nested inside region and three-way nested ANOVA for the density of fish and large free-swimming macroinvertebrate predators with ‘habitat’ (2 levels, open areas ‘O’ and submerged plants ‘S’) as an additional factor. The factor ‘lake’ nested inside region was significant for all variables (*p*<0.01).

**Table 4:** Densities of the different plant-associated macroinvertebrate taxa in the two regions.

	Mean density (±SE)		*F*-values for ANOVA factors	
	Cold Temperate	Mediterranean	Region	Lake (region)
Polychaeta	0	3.3 (3.1)	-	-
Hirudinea	103 (35)	0	-	-
Oligochaeta	9449 (2875)	450 (247)	228.3***	83.5***
Gastropoda	26.2 (8.3)	108 (59)	ns	14.4***
Malacostrada	446 (61)	703 (290)	238.4***	110.8***
Odonata	35.3 (7.8)	36.2 (14.6)	ns	15.4***
Heteroptera	4.0 (1.9)	0	-	-
Ephemeroptera	76.6 (13.1)	0	-	-
Coleoptera	7.1 (3.5)	0	-	-
Trichoptera	921 (175)	0	-	-
Diptera	12943 (3091)	10305 (1559)	11.7**	70.1***

Mean density (ind. m^−2^) of the different plant-associated macroinvertebrate taxa and results of nested ANOVA (*F*-values) on the effects of ‘region’ (two levels, cold temperate and Mediterranean and ‘lake’ (four levels) nested inside ‘region’. Significance levels:

* *p*<0.05,

** *p*<0.01,

*** *p*<0.0001,

ns, non significant (p≥0.05).

Overall, fish showed a reverse pattern to that of macroinvertebrates by exhibiting significantly higher mean densities and higher biomass in the Mediterranean than in the cold temperate region (Table 3, Fig. 1). However, as to the plant-associated macroinvertebrates, we found some significant between-lake differences (significant effect of lake in the nested ANOVA, Table 3). The fish communities in both regions were characterised by few species, most of them potential macroinvertebrate predators (Table 5). In the Mediterranean region, most of the fish species may include periphyton in their diets as well (Table 5).

**Table 5:** Densities of fish species in the lakes.

Cold temperate								
	0.3 (Lund Fjord)		0.5 (Selbjerg)		1.2 (Glombak)		3.8 (Østerild)	
	Abundance	Biomass	Abundance	Biomass	Abundance	Biomass	Abundance	Biomass
Roach ^+^ *[^35^]	5.2 (1.9)	15.1 (1.0)	37.0 (22.0)	5.8 (5.1)	2.9 (0.8)	1.4 (0.5)	0.7 (0.1)	0.1 (0.1)
*Rutilus rutilus*								
Perch ^+ ^ [^39^]	1.3 (0.3)	1.6 (0.2)	2.1 (0.5)	1.1 (0.1)	2.2 (0.9)	2.4 (0.1)		
*Perca fluviatilis*								
European smelt ^+ ^ [^35^]			0.6 (0.2)	1.7 (0.0)	0.1 (0.1)	0.3 (0.1)		
*Osmerus eperlanus*								
Three-spined stickleback ^+ ^ [^32^]			0.1 (0.1)	0.0 (0.0)	0.1 (0.1)	0.0 (0.0)	1.1 (0.3)	0.2 (0.1)
*Gasterosteus aculeatus*								
Nine-spined stickleback ^+ ^ [^32^]			0.3 (0.1)	0.0 (0.0)			0.3 (0.1)	0.1 (0.0)
*Pungitius pungitius*								
Gobiidae							0.3 (0.1)	0.0 (0.0)

Mediterranean								
	0.4 (Salins)		0.8 (Sirvent)		1.6 (Bassa Coll)		2.2 (Ter Vell)	
	Abundance	Biomass	Abundance	Biomass	Abundance	Biomass	Abundance	Biomass
Eastern mosquitofish ^+ ^ [^37^]	16.1 (5.1)	8.8 (2.4)					28.4 (8.8)	7.3 (1.0)
*Gambusia holbrooki*								
Sand smelt ^+^ * [^37]^, [38]			8.8 (3.2)	0.3 (0.1)				
*Atherina boyeri*								
Thin-lipped grey mullet ^+^ * [^39^]	0.1 (0.1)		1.0 (0.5)	0.5 (0.2)	3.6 (1.7)	1.5 (1.0)		
*Liza ramada*								
Pumpkinseed sunfish ^+^ * [^34]^, [37]	2.9 (0.7)	6.7 (1.2)	0.3 (0.1)	5.2 (1.9)	1.3 (0.3)	3.8 (1.4)	0.2 (0.1)	2.0 (0.5)
*Lepomis gibbosus*								
European eel	0.3 (0.1)	20.6 (18.2)	0.1 (0.1)	0.0 (0.1)	0.4 (0.2)	10.9 (8.0)	0.3 (0.1)	6.7 (2.6)
*Anguilla anguilla*								
Rudd ^+^ * [^36^]	0.1 (0.1)	0.0 (0.0)	0.1 (0.1)	0.4 (0.0)	0.1 (0.1)	0.1 (0.0)		
*Scardinius erythrophthalmus*								
Flathead mullet ^+^ * [^33^]							9.4 (5.7)	19.9 (8.6)
*Mugil cephalus*								
Goldfish ^+^ * [^39^]					0.1 (0.1)	0.2 (0.0)		
*Carassius auratus*								

Mean abundance (ind. m^−2^) and biomass in fresh weight (g m^−2^), with standard error (SE) of each fish species captured in the eight studied lakes and with indication of typical consumption of macroinvertebrate prey (^+^) and periphyton (*) as indicated in the literature [32]–[39].

### Changes in community structure with salinity and potential predation risk

**Figure 2:**
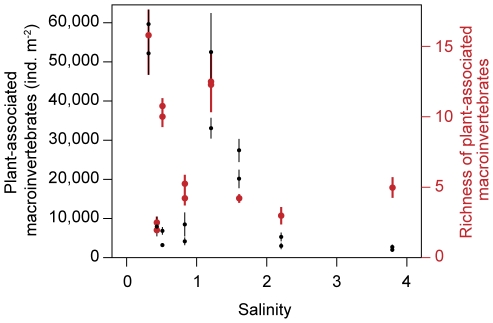
Plant-associated macroinvertebrates along the salinity gradient. Plant-associated macroinvertebrates density in black and richness in red (±1SE).

**Figure 3:**
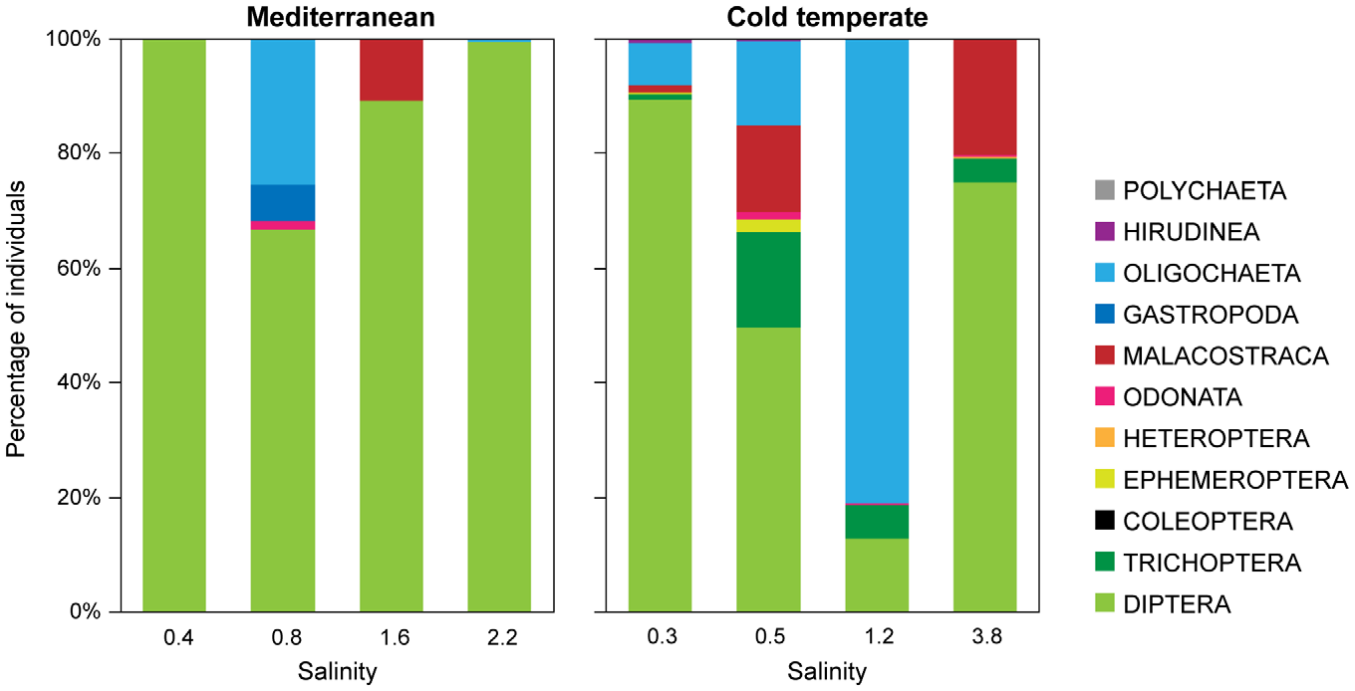
Relative abundance of plant-associated macroinvertebrate taxa.

Multiple regression confirmed a negative relationship between richness and total density of plant-associated macroinvertebrates and salinity and fish density (Table 6, Fig. 2). Most taxon groups were negatively related to salinity and fish density, except Coleoptera and Heteroptera, which were only related to salinity, Malacostraca, which was only related to fish density, and Odonata and Polychaeta, which were not related to either salinity or fish density. Nevertheless, the relative abundances of the different plant-associated macroinvertebrate taxa showed no clear trend with the salinity gradient. Diptera was the dominant taxon in all lakes except in the cold temperate lake at 1.2 salinity (Glombak), where Oligochaeta was the most abundant taxon (Fig. 3).

**Table 6:** Partial correlations from the stepwise multiple regression between the richness and density of plant-associated macroinvertebrates and the density of each taxonomic group with salinity and density of fish in that habitat (indicator of predation pressure).

Response variable	Salinity	Fish
Richness plant-associated macroinv.	−0.508***	−0.493***
Density plant-associated macroinv.	−0.653***	−0.630***
Polychaeta	ns	ns
Hirudinea	−0.584***	−0.477***
Oligochaeta	−0.635***	−0.510***
Gastropoda	−0.425**	−0.419**
Malacostraca	ns	−0.267*
Odonata	ns	ns
Heteroptera	−0.375**	ns
Ephemeroptera	−0.577***	−0.252*
Coleoptera	−0.256*	ns
Trichoptera	−0.363**	−0.478***
Diptera	−0.468***	−0.308*

Significance levels as in Table 3. Degrees of freedom of the regression and the error are 2 and 62, respectively.

**Figure 4:**
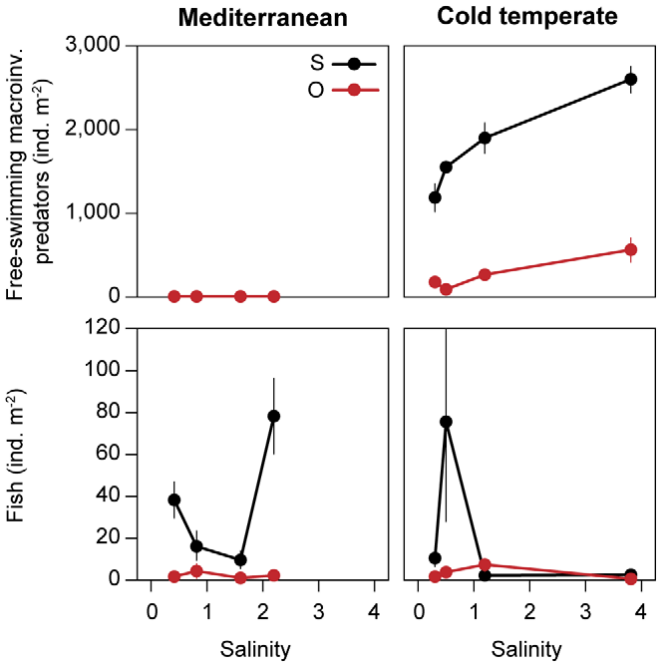
Free-swimming potentially predatory macroinvertebrates and fish at different salinities. Mean densities (mean day and night densities, ±1SE) among submerged plants (S, black) and open sites (O, red) at different salinities.

The density of free-swimming macroinvertebrates increased with salinity only in the cold temperate region (Fig. 4) and they occurred in higher abundances within the plants than at open sites (significant effect of habitat in the nested ANOVA, Table 3, Fig. 4). In the Mediterranean region, the density of free-swimming macroinvertebrate predators was very low at all four salinities (Fig. 4).

Fish density and composition showed no clear relationship with salinity and only in the Mediterranean region were the highest fish densities found at the highest salinity (Table 5, Fig. 4). Like free-swimming macroinvertebrate predators, fish were more abundant in the submerged plants beds than at the open sites in both regions (significant effect of habitat in the nested ANOVA, Table 3, Fig. 4). Only in the cold temperate region at salinity 1.2 (Glombak) and 3.8 (Østerild) did similar fish densities occur in the two habitats (Fig. 4).

### Diel distribution of organisms

**Figure 5:**
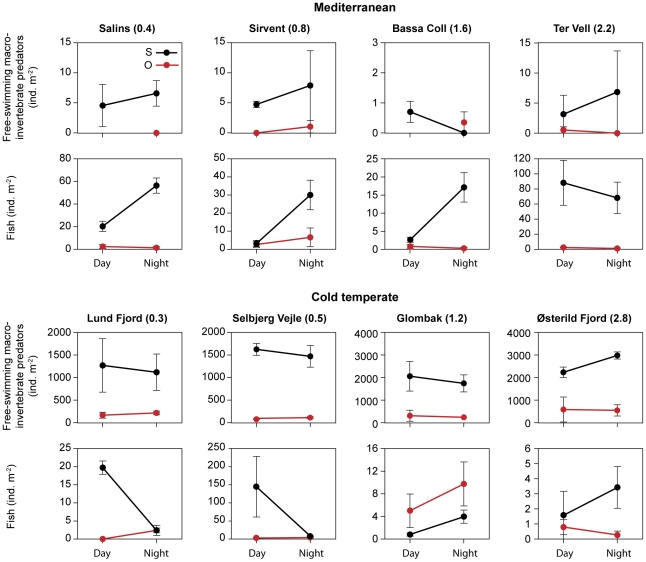
Diel changes of fish and free-swimming potentially predatory macroinvertebrates at each lake. Diel changes in density of fish and free-swimming macroinvertebrate predators between submerged plants (S, black) and open sites (O, red).

Overall, no significant differences were found between day and night-time in total abundances of plant-associated macroinvertebrates in either of the regions (Fig. 1). However, when assessing each lake separately (Fig. 5), plant-associated macroinvertebrates most frequently showed a reverse diel distribution to that of their potential predators (i.e. fish and free-swimming macroinvertebrate predators). In Denmark, higher total densities of plant-associated macroinvertebrates were found at night-time compared to daytime at 0.5 (Selbjerg) (one-way ANOVA, *p*<0.01), which was the lake with the highest fish density (Table 5, Fig. 4). In the same lake, higher abundances of Diptera and Oligochaeta were also found at night-time (one-way ANOVA, *p*<0.01 and *p*<0.05, respectively). Hirudinea was more abundant at night-time at 0.3 salinity (Lund Fjord) (one-way ANOVA, *p*<0.01). In contrast to the plant-associated macroinvertebrate diel distribution, fish were more abundant within the plants during day (two-way ANOVA, interaction between ‘habitat’ and ‘time’ p<0.01) at 0.5 (Selbjerg) and 0.3 (Lund Fjord) and free-swimming macroinvertebrate predators at 0.5 (Selbjerg) (two-way ANOVA, interaction between ‘habitat’ and ‘time’ p<0.05) (Fig. 5). The only macroinvertebrate group more abundant at daytime was Malacostraca at 3.8 (Østerild) (one-way ANOVA, *p*<0.01).

In the Mediterranean region, where fish tended to be more abundant in the plants at night [at 0.4 (Salins), 0.8 (Sirvent) and 1.6 (Bassa Coll); Fig. 5] (two-way ANOVA, interaction between ‘habitat’ and ‘time’ p<0.05), the diel distribution of plant-associated macroinvertebrates was more uniform, and only Diptera at 1.6 salinity (Bassa Coll) was more abundant at day than at night (one-way ANOVA, *p*<0.01). When present, free-swimming macroinvertebrate predators showed no significant migration pattern (Fig. 5).

## Discussion

Significant differences were found in the density and richness of the plant-associated macroinvertebrate communities in shallow brackish lakes between the cold temperate and the Mediterranean climatic regions. Overall, the plant-associated macroinvertebrates were more abundant and the communities richer in species in the cold compared to the warm climate. Diptera was the dominant taxon in both regions; however it was more abundant in the cold temperate climate, as were most of the other plant-associated macroinvertebrate taxa. Only plant-associated Malacostraca and Polychaeta had higher densities in the warm climate. The differences between regions were especially remarkable for free-swimming macroinvertebrates, which were almost absent in the Mediterranean, and for total macroinvertebrate richness, which was two-fold higher in the cold temperate climate than in the Mediterranean. The higher abundance (density and biomass) of fish in the Mediterranean region and the negative relationship between fish and plant-associated macroinvertebrate densities suggest that a higher predation pressure might be the main cause for the lower density and richness of macroinvertebrates in warm lakes, opposite to theoretical expectations [42]. In both regions, most fish species were potential predators of macroinvertebrates [36], [38], [40] and had small body sizes, as demonstrated earlier in our experiment and published elsewhere (average standard length 3 cm in both climatic regions [3], and in another study of nutrient-rich brackish lakes [43]. Heteroptera and Coleoptera were the only plant-associated invertebrates for which salinity, but not fish, had a significant effect on density. These taxa only occurred in the cold region where the gradient in fish predation pressure may be too low to detect a significant effect of fish for these taxa. Different fish species among lakes, which may have different feeding strategy and behavior [11], could also be the reason for the lake-specific differences in the abundance of plant-associated macroinvertebrates. We can furthermore not disregard potential effects of free-swimming invertebrate predators (e.g. Odonata) that may indirectly affect fish predation pressure (via competition) or directly prey upon the plant-associated macroinvertebrates. Although our results should be interpreted with caution because they are based on a small number of lakes and we did not directly control for fish abundance or predation pressure, the similarity with a previous cross-latitudinal comparison experiment in freshwater lakes [1], revealing that lower abundances of macroinvertebrates co-occur with higher abundances of fish in subtropical lakes compared to cold temperate lakes, suggests that a consistent latitudinal pattern may exist. Brucet et al. [3] had previously suggested that the higher fish predation in Mediterranean compared to cold temperate shallow lakes was the main reason for the low density of zooplankton and dominance of small sizes in warmer lakes. Thus, the role of temperature-related changes in fish predation pressure seems a key factor potentially shaping both macroinvertebrate and zooplankton communities, as also seen in shallow freshwater lakes [1].

Despite the fact that our salinity gradient was relatively small with no replication at each salinity level, salinity also appeared to have a negative effect on plant-associated macroinvertebrate richness and density. The abundance of most plant-associated macroinvertebrate taxa decreased with salinity, although no differences were found in the relative abundance of the different groups. The only taxa not related to salinity were Malacostraca, Odonata and Polychaeta, but the relative abundances of these taxon groups were low at all salinities. Diptera, one of the most salinity-tolerant groups [24], dominated in most of the lakes in the two climatic regions. The decrease in macroinvertebrate richness and abundance along the salinity gradient agrees with previous studies in lakes and ponds [23], [44] and rivers [45], [46], although some studies have shown smaller effects of salinity on total macroinvertebrate abundance due to enhanced abundances of Mollusca and Crustacea at the highest salinities [20], [47]. In the cold temperate lakes, free-swimming macroinvertebrate predators increased with salinity, which is in accordance with other studies reporting higher abundances of large pelagic macroinvertebrate predators (e.g. *Neomysis integer*) at salinities above 0.5 [15], [48]. However, this pattern was not found in Mediterranean shallow lakes, most likely as a result of the higher fish predation pressure in these lakes, although we cannot fully rule out other factors as taxa of free-swimming macroinvertebrate predators differed.

We found evidence of changes in plant-associated macroinvertebrate diel distribution for some lakes and taxa. When this occurred, plant-associated macroinvertebrates always showed a reverse diel distribution to that of their potential predators. In temperate lakes, where fish tended to aggregate within the macrophytes during day, some plant-associated macroinvertebrate taxa were generally more abundant at night than during the day, which suggests that they moved to the plants during night when fish (and also free-swimming macroinvertebrate predators) densities were lower there. This movement was apparently stronger in the lake with the highest fish densities among the plants. In contrast, in the warm lakes, where fish were more abundant within the plants at night-time or no daily differences were found, the plant-associated macroinvertebrate distribution was more uniform, and only one taxon (Diptera) was found to be more abundant at daytime. Our results thus suggest that the potential refuge of submerged plants might not counteract the effect of fish predation on macroinvertebrates to the same extent in shallow brackish lakes as in temperate freshwater lakes (see [49]), especially not in the Mediterranean lakes, because small fish are often abundant and tend to aggregate within the macrophytes.

Although we could have expected higher periphyton biomass in warm lakes as a result of lower macroinvertebrate grazing (due to the impoverished plant-associated macroinvertebrate assemblages) and better growth conditions induced by the warm climate (i.e. greater light intensity and higher temperatures), results were the opposite: average periphyton biomass was four-fold lower in the Mediterranean lakes than in the temperate lakes. A similar pattern was found by Meerhoff *et al*. [1] in their cross-latitudinal experiment with freshwater lakes showing a significantly lower periphyton biomass in the subtropical lakes than in the temperate lakes. The lower periphyton biomass found in our Mediterranean brackish lakes might be attributed to the direct (feeding) or indirect (physical disturbance) activity by the fish. Supporting this argument, the fish community in Mediterranean shallow lakes is typically dominated by omnivorous fish [38], most of which include periphyton in their diet (Table 5) to a higher extent than most fish species in north temperate lakes [6], [50]. The higher densities of Gastropoda, which are efficient grazers of periphyton [51], in some Mediterranean lakes compared to cold-temperate lakes could also promote the lower periphyton biomass in warm lakes. In Mediterranean brackish lakes, the lower periphyton biomass likely leads to reduced shading and nutrient and carbon competition with the host plants, which may eventually increase the chances of the submerged plants developing at higher turbidity levels than in comparable temperate lakes. However, although the lower density of periphyton would apparently favour macrophytes in warm lakes, the high temperature-enhanced fish predation on large-bodied zooplankton [3] may indirectly increase turbidity due to the resulting lower grazing on phytoplankton and negatively affect macrophyte growth [7], [52]. Thus, further studies are required to assess if the positive effects of the lower periphyton biomass may be counteracted by the lower grazing on phytoplankton.

In conclusion, our experiment showed marked differences in the richness and abundance of macroinvertebrate communities between cold and warm shallow brackish lakes most probably as a result of differences in fish predation pressure. Our results also suggest that where the current process of climate warming entails higher chances for shallow lakes becoming more saline, global climatic change may result in a decrease in macroinvertebrate species richness and abundance in these ecosystems. Some brackish and saline lagoons might be capable of coping with slight increases in salinity since the organisms dominating these systems can tolerate varying salinities, and well-structured communities can be found at high salinities [53]. However, as has been suggested for zooplankton [3], [17], [19], rising salinity levels, together with increasing temperatures, could have dramatic effects in slightly brackish waters (<5), whose macroinvertebrate communities comprise species from saline and freshwaters, and even more severe impacts on current freshwater lakes.
